# Traumatic Bilateral Brachial Plexus Injury

**DOI:** 10.7759/cureus.24626

**Published:** 2022-04-30

**Authors:** Zinon Kokkalis, Spyridon Papagiannis, Antonis Kouzelis, George Diamantakis, Andreas Panagopoulos

**Affiliations:** 1 Orthopaedics and Traumatology, Patras University Hospital, Patras, GRC

**Keywords:** brachial plexus neuropathies, oberlin procedure, upper extremity trauma, bilateral brachial plexus injury, brachial plexus injury

## Abstract

Traumatic brachial plexus injuries are serious, life-changing injuries that are becoming more common worldwide. A thorough physical examination, as well as radiologic and electrodiagnostic tests, are all part of the initial evaluation. Parameters such as injury patterns, the timing of intervention, patients' expectations, and pre-injury functional level should always be considered. A bilateral brachial plexus injury is a very uncommon occurrence. To our knowledge, only one case of a bilateral brachial plexus injury associated with trauma has been published in recent literature. We present a rare case of a 19-year-old man who sustained a bilateral brachial plexus injury after a motorbike accident. The patient underwent exploration of the left brachial plexus and a modified Oberlin procedure on his left arm. The right plexus injury was managed conservatively. After a follow-up period of 12 months, the patient completely returned to his previous functional level.

## Introduction

The brachial plexus is a complex neural network that innervates the arm, shoulder, and upper chest via motor and somatosensory nerves. The brachial plexus is typically made up of the ventral rami of the C5-T1 spinal nerves. These ventral rami represent the roots of the plexus that combine to generate three trunks, six divisions, three cords, and five terminal motor/sensory branches to the upper extremities. In around half of the cases, however, the brachial plexus anatomy can be a variant [1]. As a result, different injury patterns can lead to diverse neurological abnormalities. A bilateral brachial plexus injury is a rare entity, with cases reported in literature associating this condition with prolonged use of crutches, intraoperative shoulder bracing, and complications during childbirth. However, only one case of bilateral traumatic plexopathy has been reported [2]. In an attempt to elucidate the potential mechanisms and demonstrate our therapeutic approach, we present an unusual case of a 19-year-old man who incurred a bilateral brachial plexus injury after a motorcycle accident.

## Case presentation

**Figure 1:**
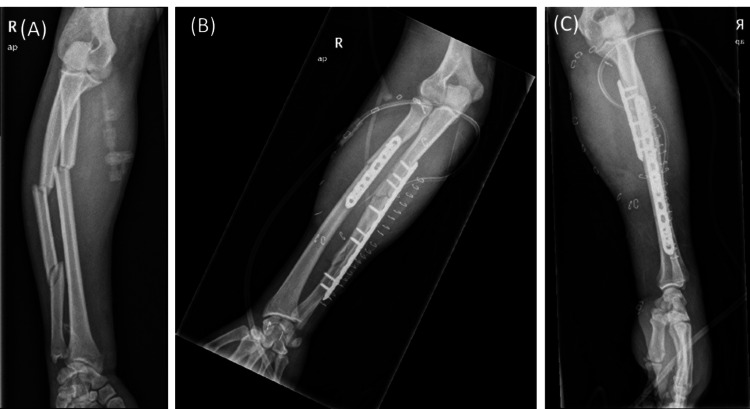
(A) Preoperative X-ray of a right forearm fracture, (B, C) Postoperative X-rays

**Figure 2:**
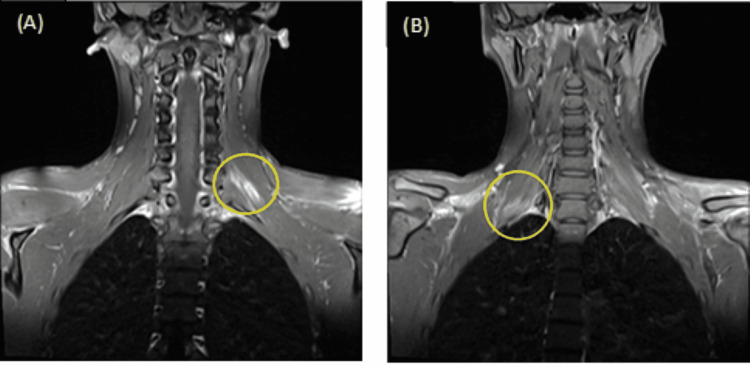
(A): MRI image showing swelling of the C6, C7, and C8 nerves and hematoma in the surrounding tissues of the left brachial plexus (inside the yellow circle), (B): MRI image showing thickening of the C6, C7 and C8 nerves of the right brachial plexus (inside the yellow circle)

The patient, a 19-year-old man, was transferred to the Accident and Emergency department of our hospital after a motorbike accident. He was a non-smoker, with no medical comorbidities. He was initially managed according to the Advanced Trauma Life Support (ATLS) guidelines. At initial evaluation, the patient was unable to move his shoulders, elbows, and wrists. Contraction was present on the elbow and wrist flexors and extensors on both upper limbs, with a Medical Research Council scale (MRC scale) score of 1/5 on both arms. Finger flexion and extension were partially limited on both arms with an MRC scale score of 3/5 bilaterally. The patient was maintaining sensation in both upper extremities and the vascular system was intact. Swelling was present on the right forearm, and a 10 cm laceration on the medial side of the patient’s left cubital fossa was identified. Initial X-rays showed a right forearm fracture classified as 22C1 according to the Muller AO classification (Figure 1A) [3]. There were no other concomitant injuries, and the patient was admitted to the orthopedic ward. The next day, open reduction and internal fixation of the right forearm fracture were performed (Figures 1B-1C), with no nerve injury identified intraoperatively. The ulnar incision was partially closed using a fasciotomy equivalent because of excessive edema. The left cubital fossa trauma was irrigated, debrided, and closed at the same time, without detecting any neurovascular damage. Postoperatively, no improvement in the MRC scale in both upper limbs was identified. On the fourth postoperative day, the patient was rescheduled for ulnar trauma closure and a thorough surgical exploration was performed. No signs of nerve injury were observed. A cervical spine and bilateral brachial plexus MRI with intravenous contrast injection revealed swelling of the C6, C7, and C8 roots of the left brachial plexus with possible disruption of their route and hematoma in the surrounding tissues (Figure 2A). Thickening of the C6, C7, and C8 nerves was identified on the right brachial plexus (Figure 2B).

**Figure 3:**
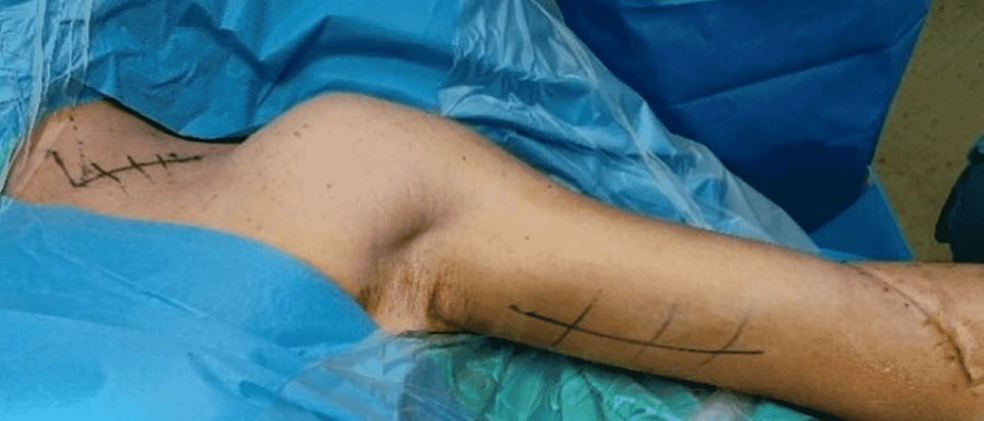
Patient’s position and planning of the two skin incisions for left brachial plexus investigation and modified Oberlin procedure

**Figure 4:**
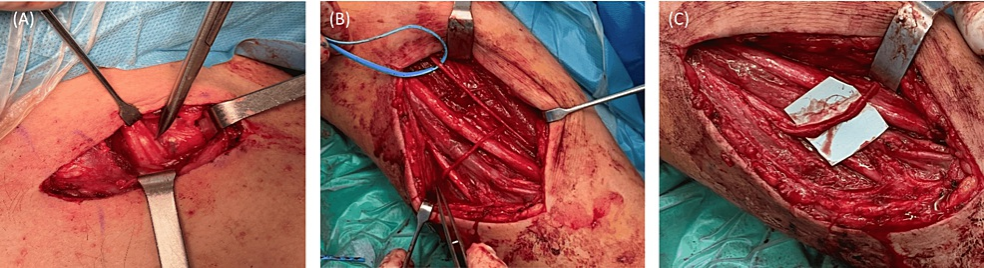
(A) Nerve roots were intact and mild swelling was present. External neurolysis was performed in the upper and middle trunks, (B): Musculocutaneous and ulnar nerves were identified and dissected, (C): A modified Oberlin procedure was performed. The brachialis motor branch was dissected, and its distal end was sutured with two ulnar nerve fascicles

The right plexus injury was managed conservatively. The left brachial plexus deficit was surgically treated one month after the initial injury. The patient was placed in a supine position with the arm in lateral abduction and a supraclavicular incision was performed (Figure 3). A nerve stimulator (Vari Stim® III, nerve locator, Medtronic Xomed, Inc., Jacksonville, FL) was used during the procedure to access proper nerve identification and function. Exploration at the supraclavicular area revealed only swelling of the brachial plexus roots with no signs of rupture. External neurolysis was performed in the upper and middle trunks (Figure 4A). A modified Oberlin procedure was performed for restoration of elbow flexion [4]. A longitudinal incision was made 4 cm distal to the humeral insertion of the pectoralis major tendon, along the medial aspect of the left arm. Between the biceps and coracobrachialis muscles, the musculocutaneous nerve was reached and the nerve branches to the biceps and brachialis were recognized. Some contraction of the biceps was identified using the nerve stimulator but contraction of the brachialis muscle was absent. The decision was to neurotize only the branch to the brachialis muscle. The ulnar nerve was dissected at the same level and two ulnar nerve fascicles that innervated the flexor carpi ulnaris were dissected. The brachialis motor branch was dissected, and its distal end was twisted medially toward the ulnar nerve. Two ulnar nerve fascicles were chosen and isolated from the rest of the ulnar nerve and then rotated laterally and sutured to the brachialis motor branch with nylon 9-0 suture (Figures 4B-4C).

**Figure 5:**
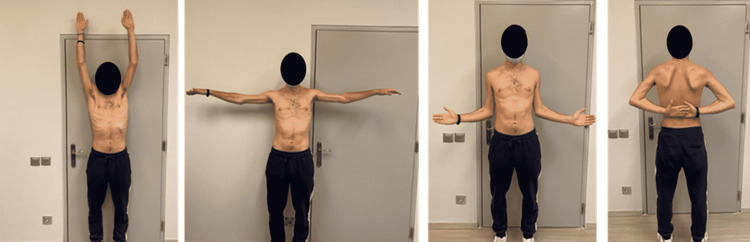
Full range of motion present at the 12-month follow-up

There were no postoperative complications and the patient was discharged four days after surgery. An intensive rehabilitation program began from the first postoperative week including range-of-motion exercises of the fingers along with transcutaneous electrical nerve stimulation (TENS). After a period of three months, muscle movement against gravity without any resistance (3/5 on the MRC scale) was present on both upper limbs. At the four-month follow-up, the patient's MRC scale score was 4/5. Finally, at the 12-month follow-up, unrestricted range of motion was present (5/5 on the MRC scale) on both upper limbs. The patient was able to return to his pre-injury level of function (Figure 5) with a Disabilities of Arm, Shoulder, and Hand (DASH) score of 2.5 at that time.

## Discussion

Brachial plexus injuries are uncommon, with a prevalence of 1.2% in multi-trauma patients [5]. The Swiss study by Narakas [6], who described the ‘rule of 7x70%’, has traditionally been the standard reference for the etiopathogenesis of brachial plexus injuries. Seventy percent (70%) of brachial plexus lesions were associated with motorcycle or bicycle accidents. Seventy percent (70%) of the cases were polytrauma patients presenting with supraclavicular lesions. At least one root avulsion was identified, with avulsions mostly affecting the lower plexus. Chronic pain was present in 70% of the patients. From an initial dominance of open injuries to the current majority of closed lesions, the etiology of brachial plexus injury has changed. Direct pressure, traction, compression, recurrent microtrauma, and compression or stretch-induced ischemia are some of the potential mechanisms. The main cause of closed injuries is motorcycle accidents while Jain et al. mentioned that the dominant limb is affected more frequently [7]. When the limb is along the chest and the force acts from above, the supraclavicular upper brachial plexus injuries are more possible. The elements of the lower plexus are at risk if the arm is aggressively dragged into abduction and extension. Complete lesions are caused by significant forces or a combination of numerous forces. The upper roots have a significantly higher proclivity for rupture since they are protected from avulsion by dural sleeves and fibrous connections. On the contrary, lower plexus injuries are generally caused by the avulsion of C8-T1 roots [8]. Motor roots are more prone to rupture because they are thinner than sensory roots [9]. Although brachial plexus injuries have been well-described, traumatic bilateral brachial plexus lesions remain a rare and elusive condition. Ramdass et al. reported a case of bilateral traumatic brachial plexus injury in a 33-year-old patient after a motorbike accident [2]. In their case, the possible mechanism of injury was traction of the head and forced lateral flexion of the neck. The patient was treated conservatively. Although there was some gradual improvement, the patient retained a characteristic posture with both arms hanging and internally rotated. Bilateral brachial plexus neuropathy has been associated with non-traumatic conditions such as prolonged use of crutches [10], direct plexus pressure caused by immobilization during surgical interventions [11-13], and complications during childbirth [14]. In our case, traction, compression, and direct forces applied to the patient’s shoulder and neck at the time of injury were potential causes of traumatic bilateral plexus lesions. Meanwhile, the patient’s position after the accident and until the time of extrication may predispose them to bilateral plexus neuropathy caused by direct pressure.

In our case, there was high clinical suspicion of bilateral plexus injury based on the patients’ clinical presentation. The MRI revealed multiple root swellings on both sides, with possible disruption in the left brachial plexus. Edema in the right plexus roots was less significant and a gradual improvement in the patient’s right upper limb muscle strength was observed (from 1/5 to 2/5 on the MRC scale), one month after the injury. Thus, we decided to manage the right plexus injury conservatively. No signs of recovery were identified in the left upper limb one month after the initial injury, with an MRC scale score for elbow and wrist flexors and extensors of 1/5. Patients suffering from stretch and blunt injuries of the brachial plexus are treated conservatively initially. A three-month period of observation can reveal evidence of muscle regeneration. Surgery is recommended in the absence of spontaneous healing after three months [15]. Although our patient was fully informed about the possibility of spontaneous healing after a three-month period, he was unwilling to wait. Based on the patient's age and expectations for earlier results along with no signs of recovery at one month, we decided to operate earlier than indicated. External neurolysis and nerve transfer at the left brachial plexus offered the patient an excellent result, and he was able to return to his previous level of activity.

## Conclusions

A bilateral brachial plexus injury is a very uncommon but extremely significant medical condition. A high level of suspicion should arise when managing polytrauma patients, especially after motor vehicle accidents. Meticulous clinical examination, MRI, and electrodiagnostic tests are the main diagnostic tools. Understanding the etiopathogenesis and mechanisms behind this complex entity can help in choosing the most appropriate treatment.
